# A multicenter observational cohort study in survivors of Down Syndrome-associated acute leukemia (ALTE22C1): a report from the Children’s Oncology Group

**DOI:** 10.1186/s12885-025-14898-z

**Published:** 2025-10-20

**Authors:** M. Monica Gramatges, Lauren N. Sanclemente, Lacey Hall, Olga A. Taylor, Michelle M. Nuño, Smita Bhatia, Eric J. Chow, Kelly D. Getz, Johann K. Hitzler, Amanda M. Li, Kaitlin McCloskey, Paul C. Nathan, Maureen M. O’Brien, Serina Patel, Anupam Verma, Angela R. Yarbrough, Melissa A. Richard, Tracie C. Rosser, Lisa M. Jacola, Philip J. Lupo, Karen R. Rabin

**Affiliations:** 1 Texas Children’s Hospital, Baylor College of Medicine, 6620 Main St., Ste 1530, Houston, TX 77030 USA; 2 St. Jude Children’s Research Hospital, Memphis, TN USA; 3 Children’s Oncology Group, Monrovia, CA, University of Southern California, Los Angeles, CA USA; 4 University of Alabama at Birmingham, Birmingham, AL USA; 5 Fred Hutchinson Cancer Center, Seattle Children’s Hospital, University of Washington, Seattle, WA USA; 6 University of Pennsylvania, Philadelphia, PA USA; 7 The Hospital for Sick Children, Toronto, ON Canada; 8 BC Children’s Hospital, University of British Columbia, Vancouver, BC Canada; 9 University of Michigan, Ann Arbor, MI USA; 10 Children’s Hospital Colorado, University of Colorado Anschutz Medical Campus, Aurora, CO USA; 11 Children’s Hospital, London Health Sciences Centre, London, ON Canada; 12 Pediatric Specialists of Virginia, Fairfax, VA USA; 13 Children’s National Hospital, Washington, DC USA; 14 The University of Texas MD Anderson Cancer Center, Houston, TX USA; 15 Emory University, Atlanta, GA USA; 16 University of California-San Francisco, San Francisco, CA USA

**Keywords:** Down syndrome, Childhood leukemia, Cancer survivorship

## Abstract

**Background:**

Down syndrome (DS) is a common genetic disorder resulting from an extra copy of genetic material from all or part of chromosome 21. Individuals with DS have a higher burden of co-occurring structural birth defects, neurocognitive delay, and chronic health conditions when compared to those without DS, as well as a 10 to 20-fold excess risk for acute leukemia (AL). Few studies have reported the late effects of cancer treatment in DS-AL survivors, and even fewer have compared outcomes to children with DS and no cancer history. The Children’s Oncology Group study ALTE22C1 was developed to address this knowledge gap.

**Methods:**

This study leverages both registry and site-based DS-AL survivor recruitment and a prospective/retrospective cohort design to compare chronic health conditions and neurocognitive outcomes experienced by DS-AL survivors to age and sex-matched individuals enrolled to a DS cohort for which cancer is exclusionary. Survivors 6–39 years old, ≥ 3 years from end of AL treatment, and in remission are eligible. Participants complete a medical conditions survey and neuropsychological battery by parent proxy and may also participate in an in-person physical and neurocognitive assessment. Biological samples are collected to evaluate molecular features associated with outcomes.

**Discussion:**

This cooperative group study will identify the prevalence and severity of medical and neurocognitive outcomes in DS-AL survivors compared with non-DS AL and DS controls without cancer history. Results are anticipated to inform clinical practice guidelines for DS-AL survivors and improve survivor outcomes through mitigation of outcome disparities in this vulnerable population.

**Trial registration:**

Children’s Oncology Group study ALTE22C1 is registered under the ClinicalTrials.gov identifier NCT05702645.

## Background

Survival from childhood and adolescent cancer has increased significantly in the past several decades, from under 50% in the 1970s to over 80% today [1]. This remarkable achievement has resulted in an exponential increase in the number of childhood cancer survivors living in the United States, projected to exceed 500,000 by this year [2]. However, the price of cure includes a significant burden of treatment-related morbidities. Seventy percent of adult survivors are estimated to suffer from one or more severe, disabling, or life-threatening chronic health conditions (CHC) or 'late effects' that significantly and negatively impact their quality of life [3, 4]. Down syndrome (DS) is a genetic disorder that results from a constitutional trisomy of chromosome 21. This disorder is associated with distinguishing phenotypic features, intellectual disability, co-occurring structural birth defects, and an increased risk for CHC. Children with DS are also at increased risk for developing hematologic malignancies, with a 10 to 20-fold excess risk in comparison with the general population, so that between 1:100 and 1:150 children with DS will develop acute leukemia (AL), [5, 6] predominantly B-lineage acute lymphoblastic leukemia (ALL) or acute myeloid leukemia (AML), composing 2% of all pediatric ALL cases and 15% of all pediatric AML cases. During treatment for AL, children with DS experience excess acute cancer therapy-related toxicities compared to their non-DS peers with ALL and AML [7–9].

In the absence of DS, AL treatment confers a substantial risk for late effects. Among 272 AML survivors enrolled to the Childhood Cancer Survivor Study (CCSS), the cumulative incidence of CHC at 20 years from diagnosis was 50%, of which 16% were severe, compared with only 6% of sibling controls [10]. Similarly, at 25 years from diagnosis, the cumulative incidence of CHC among ALL survivors enrolled to CCSS was 65%, of which 21% were graded as severe [11]. A meta-analysis of neurocognitive sequelae of childhood ALL provides consistent evidence that cancer therapy impacts intellectual function even in the absence of radiation exposure, particularly with respect to attention, working memory, and processing speed [12, 13], although less is known regarding the neurocognitive sequelae of AML treatment [14].

Despite the well-described late effects associated with childhood AL treatment and the known excess acute toxicities experienced by children with DS-AL, little is known regarding the prevalence of long-term therapy-related CHC and neurocognitive outcomes in survivors of DS-AL. As a result, the National Down Syndrome Society and Lumind, IDSC identified the impact of leukemia and its therapy on neurodevelopmental, health, and quality of life outcomes as one of the key scientific gaps in DS research [15].

The Children’s Oncology Group (COG) study ALTE22C1 (ClinicalTrials.gov Identifier: NCT05702645) was developed to address this unmet need, with the overarching hypothesis that DS-AL survivors are at increased risk for CHC and neurocognitive deficits compared to persons with DS and no cancer history.

## Methods/design

### Aims

The primary aim of this study is to determine the prevalence, type, and severity of CHC in survivors of DS-AL and to compare CHC outcomes to those of frequency-matched individuals with DS with no cancer history. Secondary aims include characterizing post-treatment clinical outcomes of DS-AL, determining prevalence and severity of parent-reported neurocognitive outcomes in survivors of DS-AL compared with frequency-matched individuals with DS without leukemia, determining health-related quality of life in survivors of DS-AL, and identifying clinical risk determinants of CHC, neurocognitive outcomes, and clinical outcomes in survivors of DS-AL. Biological and molecular correlations will be tested in exploratory aims, i.e., we will test whether structural birth defects and genetic alterations associated with etiology also demonstrate associations with CHC in DS-ALL, and whether telomere length is associated with in-person-assessed neurocognitive outcomes in DS-ALL.

### Study design

**Fig. 1:**
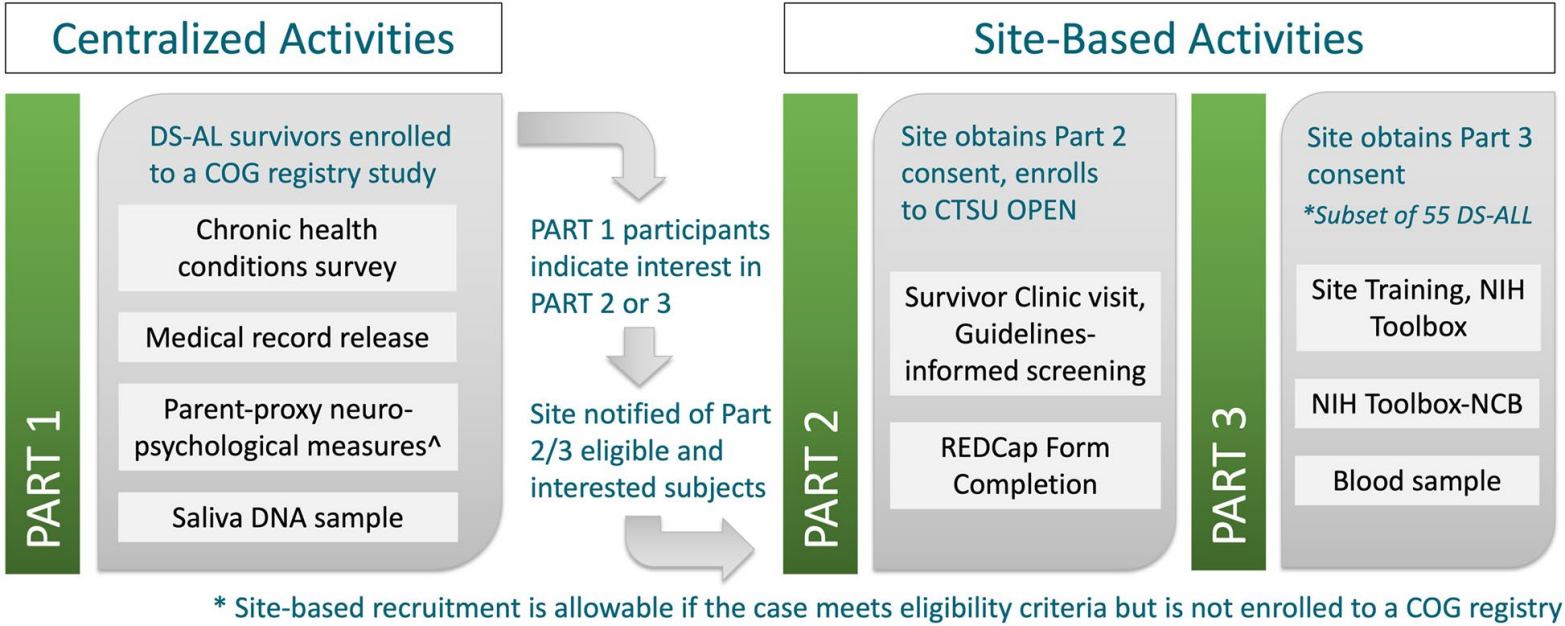
Study Design Schema for COG ALTE22C1. ^See Table 1 for list of measures

Children’s Oncology Group ALTE22C1 utilizes a retrospective/prospective observational cohort design with a control group comparison. For survivors of AL, study participation is divided into three parts, with a workflow that relies on centralized Data Coordinating and Neuropsychology Centers to minimize responsibilities of COG site-based research staff. Study participants, or his/her designee, provide separate consent for participation in Parts 1, 2, and 3. See Fig. 1 for the study design schema.

**Fig. 2:**
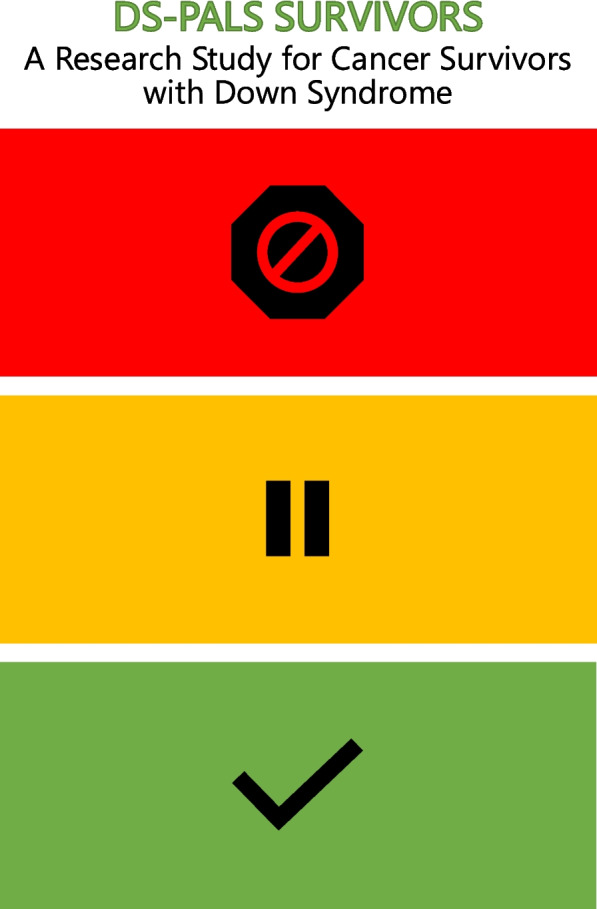
Visual Break Board. This visual break board is adapted from Kidney, et al., 2014 and is available to participating sites to facilitate subject recruitment and promote inclusion in research of neurodivergent populations. The following instructions are provided to support its use. *How to use this tool*: Prior to initiating a consent discussion, provide the patient with this ‘stoplight’ sheet or card. Advise him/her that you will be discussing a research study and would like to make sure that he/she is comfortable and understands what is being said. Let the patient know that he/she may indicate Red, Yellow, or Green any time during the discussion to indicate: • ‘Stop, I would like to end this conversation,’ • ‘Pause, I need a break,’ or • ‘I’m OK, please keep going’• Pause periodically and prompt the patient to use the board to provide you with an indication to stop, pause, or go on.

Part 1 is conducted remotely through REDCap® [16, 17] (with a paper alternative) and consists of a medical record release, medical condition survey (completed by parent proxy and verified by medical record review), and saliva sample. Part 1 also includes a neurocognitive battery completed by parent proxy and designed to evaluate executive function and self-regulation, social competence, problem behaviors, autism spectrum behavior, communication, adaptive behavior, and quality of life. The measures included and their target area of assessment are shown in Table 1 [18–23]. Part 2 is focused on in-person assessments and requires local COG site activation of this central-IRB-approved protocol. For Part 2, consent is obtained by COG site personnel. Resources are available to sites that support inclusion in research of persons with intellectual disabilities [24], including a visual break board for survivors to direct the pace of the consent conversation and request a pause or stop if needed (Fig. 2) [25]. Part 2 comprises an in-person, COG Long-Term Follow-Up Guidelines [26]-informed and Down Syndrome Guidelines [27, 28]-informed long-term follow-up visit. Results of the clinic visit, including diagnosis and treatment information, complete review of systems, vital signs and anthropometrics, Tanner staging, list of medication, and results of any laboratory or diagnostic tests, are reported by the COG site-based research team via REDCap® to the central data coordinating center. Part 3 comprises a comprehensive, in-person neurocognitive evaluation on a subset of ALL survivors and research blood draw, also requiring COG site activation in addition to COG site-based training. The COG site-based research team administers the NIH Toolbox Cognitive Battery (NIH-CB) using a study-supplied tablet. The NIH-CB is validated in persons with intellectual disability [29, 30] and includes seven tests: Flanker Inhibitory Control and Attention, Dimensional Card Sort, List Sorting Working Memory, Oral Reading Recognition, Pattern Comparison Processing Speed, Picture Sequence Memory, and Picture Vocabulary. The Part 3 required research blood draw is banked for future assessment of leukocyte telomere length by telomere flow-fluorescence in situ hybridization (FISH) [31].

**Table 1:** Part 1 and Part 3 Neurocognitive Measures Included in COG ALTE22C1

Neurocognitive Measures	Abbreviation	Target Assessment
Part 1: Parent Proxy		
Behavior Rating Inventory of Executive Function, 2nd ed	BRIEF-2	Executive functioning and self-regulation
Vineland Adaptive Behavior Scales, 3rd ed	Vineland-3	Adaptive behavior
Social Communication Questionnaire	SCQ	Autism spectrum disorder screening
Nisonger Child Behavior Rating Form	NCBRF	Social competence and problem behaviors
Children’s Communication Checklist, 2nd ed	CCC-2	Communication and language impairment screening
Pediatric Quality of Life Inventory, Generic Core Scales v4.0	PedsQL	Health related quality of life
Part 3: Direct Assessment		
NIH Toolbox Cognitive Battery	NIHTB-CB	Executive function: inhibitory control, cognitive flexibility, attention

### Study setting

This study leverages the COG cooperative group infrastructure. The COG is a member of the National Cancer Institute-funded Community Oncology Research Program and the National Clinical Trials Network, which includes over 220 academic and community hospitals that care for ~ 90% of children with cancer diagnosed in the United States [32].

### Recruitment, population, and eligibility

A unique feature of this observational survivorship trial is the hybrid recruitment approach that leverages the COG Childhood Cancer Research Network (ACCRN07) and Project EveryChild (APEC14B1) research registration and biobanking protocols [33, 34]. Potential participants with DS-AL are identified and recruited by two mechanisms run in parallel: 1) The Coordinating Center conducts centralized recruitment of patients that have consented to recontact and were enrolled to either ACCRN07, APEC14B1, and/or AEPI20N2 The DS-ALL Deep Phenotyping Project: Evaluating the Association Between Co-occurring Birth defects and ALL Risk in DS; 2) Site-based prospective recruitment at COG sites that have activated the study protocol (only for subjects not enrolled to any of the above registries or studies).

Eligible survivors are those with DS-AL who are ≥ 6 years old and < 40 years old, in remission, at least 36 months off primary cancer treatment, and able to understand either English or Spanish. Bone marrow transplant recipients are excluded (see Table 2). A diagnosis of Down syndrome is required and may include any of the three recognized types: trisomy 21 resulting from chromosomal nondisjunction (most common), translocation (the patient has 46 chromosomes, but all or part of an additional copy of chromosome 21 is attached to another chromosome), or mosaicism (trisomy 21 that is present in only a fraction of cells). All participants to ALTE22C1 must be a survivor of either ALL or AML/myeloid leukemia of DS (ML-DS) requiring treatment with chemotherapy and may not have had any other cancer diagnosis other than transient abnormal myelopoiesis. Based on the racial and ethnic distribution of childhood leukemia in the United States, the sample is expected to be 70% White, 10% Black, 20% other or multi-race, and about 20% Hispanic (estimates derived from the Surveillance Epidemiology and End Results Program).

**Table 2:** Eligibility Criteria for COG ALTE22C1

Inclusion Criteria
• Age ≥ 6 and < 40 years at the time of enrollment
• Diagnosis of Down syndrome
• Survivor of ALL or AML treated with chemotherapy for their cancer diagnosis
• Completed treatment at least 36 calendar months prior to enrollment
• Life expectance > 1 year
• English or Spanish speaking
Exclusion Criteria
• History of hematopoietic stem cell transplant
• History of another cancer either prior to or subsequent to the ALL/AML diagnosis
• Parent or guardian unable to complete the required forms

The comparison population (‘DS controls’) are active participants in an ongoing research study cohort of children and young adults with DS. Recruitment to this study began in 1989 as a population-based case–control study conducted in the five-county metropolitan area of Atlanta and has since expanded recruitment to sites across the United States. Recruitment and enrollment methods to this study are described previously [35–38]. Controls will be frequency-matched to cases by age, sex, and, when possible, race and ethnicity.

### Role of coordinating centers

The Data Coordinating Center recruits and consents to Part 1 study activities and provides participants with Part 1 study materials. The plan for patient approach is first communicated and cleared through the COG institution of record in the form of an email notification that includes the affiliated subject’s COG ID number. The site has an opportunity to decline or postpone subject approach: 14 calendar days following the date of site notification, if no communication of concern has been received from the site, the Data Coordinating Center mails the recruitment packet directly to the survivor’s email or physical address followed by a phone call check-in approximately two weeks later. In addition, the Data Coordinating Center tracks all potential and enrolled participants, and, for enrolled participants only, ensures that study data and samples are collected in a timely fashion and that participants receive a reimbursement associated with the completion of each of the three study parts. The role of the Neuropsychology Coordinating Center is to ensure that Part 1 neurocognitive assessment data are collected and are complete, as well as to interpret results and prepare and return an abbreviated summary of the results to the family. The Neuropsychology Coordinating Center also provides the COG site with Part 3 NIH Toolbox training in addition to Part 1 neurocognitive test result interpretation.

### Study outcomes

The primary outcome is occurrence of CHC by category, organ system, and grade using the Common Terminology Criteria for Adverse Events (CTCAE), for both DS-AL survivors and DS controls. Secondary outcomes include clinical outcomes from the LTS visit, with laboratory and diagnostic tests categorized as ‘normal’ or ‘abnormal,’ and neurocognitive outcomes measured by standardized caregiver ratings (raw and standardized score as well as proportion ‘at the floor’ in each comparison group). Exploratory outcomes include raw and standardized scores as measured by the NIH Toolbox-neurocognitive battery.

### Sample size and power calculations

In a pilot study, we observed a higher likelihood of acquired cataracts among DS-AL survivors compared to DS controls, possibly related to steroid exposure during leukemia treatment (p-value = 0.03, all cases were DS-ALL survivors treated with steroids), and a higher likelihood of any cardiovascular or cerebrovascular event among DS-AL survivors compared to DS controls, possibly related to anthracycline exposure during leukemia treatment (p-value = 0.01, all cases were anthracycline-exposed). Based on these differences, with a sample size of 200 DS-ALL survivors and 200 DS controls, we will have 80% power to detect a hazard of 0.046 or 0.084, assuming a 2-sided alpha of 0.05 and an exponential baseline hazard of 0.062. With a sample size of 100 DS-AML survivors and 100 DS controls under the same assumptions, we will have 80% power to detect a hazard of 0.040 or 0.096. Based on the findings of Roncadin et al., [39] we will have 90% power to detect a 10-point difference in mean score for adaptive behavior. Assuming an expected standard deviation (SD) of 20 based on published data using the PedsQL to assess quality of life in populations with intellectual disabilities in comparison to those without disabilities [40], we will have 80% power to detect a difference in the mean scores of at least 4 to 6 points between DS-AL survivors and DS controls, sufficient to permit differentiation of healthy from acute or chronically ill subjects [41].

### Data collection, classification and handling of missing or incomplete data

Part 1 measure responses will be securely transmitted electronically after completion by parent proxy to a central study database or manually entered for any responses on paper. Part 1 CHC data, including date of onset, are abstracted, categorized by organ system, and graded using the CTCAE v5.0 at the Data Coordinating Center, using previously described methods for identifying CHC in childhood cancer survivors [42]. Part 1 neurocognitive data are scored at the Neuropsychology Coordinating Center, with final scores entered in REDCap. Part 2 clinical outcome results are securely transmitted electronically from the COG site to a central study database and categorized by the Data Coordinating Center as ‘normal’ or ‘abnormal.’ Any missing tests recommended by clinical practice guidelines are noted as ‘incomplete.’ The availability of in-home, remote completion, loaner tablets, and mobile Wi-Fi hotspots, in addition to compensation, are expected to facilitate data collection and reduce data missingness. Results from parent proxy neurocognitive assessments are summarized and provided to families at no cost to further incentivize participation and to facilitate subject access to special needs services.

For DS control participants, outcome data are collected using identical measures to assess CHC in addition to parent proxy completion of an identical neurocognitive battery. Similar to DS-AL cases, medical outcomes are verified by medical record review after a medical release is obtained, including both birth defects and CHC (type, severity, date of onset). DS controls do not complete the NIH Toolbox assessment and do not submit a biological specimen.

### Statistical analyses

For Part 1 data, the prevalence for each CHC will be estimated and compared with published CHC prevalence and grading data from non-DS AL survivor cohorts using CTCAE criteria [10, 11, 43, 44]. We will then construct longitudinal data to power time-to-event analyses, using two modeling approaches [45] to account for time-varying independent variable (diagnosis with AL) effects on time to a dependent variable (CHC incidence and CHC-free survival). Subjects without a CHC event will be censored at the time of study enrollment (DS-AL) or update of CHC or neurocognitive data (DS controls). For all analyses, we will pool observations over equal length intervals of chronologic age to predict short term risk of CHC during each age interval. First, we will use pooled logistic regression to estimate OR, 95% CI, and p-values for association of ALL, AML, or any AL diagnosis with medical record-verified CHC, agnostic of time to CHC. Next, we will use stratified Cox models to refine associations of ALL, AML, or any AL diagnosis with CHC based on time to CHC incidence. Within each age interval, we will estimate the hazard ratios (HR), 95% CI, and p-values to report time-dependent effects of ALL, AML, or any AL diagnosis on CHC. All models will be adjusted for age, sex, race/ethnicity, and relevant cancer or DS characteristics.

For Part 1 neurocognitive data, we will determine both raw and standardized scores derived from age-based norms for all tests. Quantitative data will be summarized with descriptive statistics and correlational techniques. Given inherent challenges of interpreting age-normalized results in an intellectually disabled population, data will be evaluated for evidence of floor effects, i.e., an atypical left-shifted distribution of standard scores for measures where lower scores suggest lower skill ratings (Vineland, CCC-2) or a rightward shift for measures where higher scores indicate more behavioral issues (BRIEF-2, SCQ, NCBRF). Mean scores for each assessment will be compared between DS-AL survivors and DS controls, as well as between DS-AL subgroups and DS controls. The mean score for each test will be compared between the cohorts using a Student t-test (2-sided significance level of 0.05 and equal variance). If atypical score distributions restrict our ability to use parametric statistics, we will consider nonparametric approaches to group comparisons or score transformations to facilitate use of parametric modeling. In addition, we will utilize frequency comparisons, with scores dichotomized as clinically significant impairment yes/no according to manualized procedures (≤ 1.5 SD outside of the mean for the age-normative sample) and will compare the proportion of subjects ‘at the floor’ between DS-AL survivors, DS-AL subgroups, and DS controls.

For Part 2, the proportion of results that are ‘abnormal’ for each test will be detailed using descriptive statistics and compared between DS-ALL and DS-AML survivors.

For Part 3, we will use Cox regression to estimate the HR and 95% CI for number, severity, and type of CHC, as well as for neurocognitive deficits dichotomized as clinically significant impairment yes/no (≤ 1.5 SD outside of the mean for the age-normative sample) according to age at diagnosis, years off therapy, sex, race/ethnicity, diagnosis, and treatment exposures. We will use Cox regression to estimate HR and 95% CI for number, severity, and type of CHC by number of birth defects, as well as explore the role of DS-ALL susceptibility variants identified in our genome-wide assessment [46] on DS-associated CHC in survivors. All models will be adjusted for sex, genetic ancestry (estimated using the top 10 principal components), and age at diagnosis (years). We will determine genetically-estimated telomere length (TL) using methods described previously [47] and use a multivariable Cox proportional hazard model to estimate HR, 95% CI for the association between each of nine variants and risk for neurocognitive deficit ≤ 1 SD below the mean, considering the weighted and unweighted risk score from the number of risk alleles present and previously-published beta estimates. For flow-FISH analyses, TL ≤ 1 st percentile is considered ‘very short,’ as previously defined [48]. We will determine ORs for neurocognitive deficits ≤ 1.5 SD below the mean for a DS age-normative sample in subjects with TL > or ≤ 1 st percentile, adjusted for relevant covariates and treatment factors (e.g. ALL, AML). We will report 95% CI, p values using McNemar’s test (two-tailed p-value of 0.05 statistically significant).

### Study progress

ALTE22C1 opened for recruitment in June 2023. As of August 2025, 55 COG sites have activated the protocol and 112 participants have been enrolled to Part 1, 73 to Part 2, and 6 to Part 3.

## Discussion

ALTE22C1 is an ongoing multisite study open through the COG cooperative group that will compare the CHC and neurocognitive outcomes in DS-AL survivors, compared to persons with DS and no cancer history. The hybrid recruitment design leverages both COG patient registries as well as site-based recruitment of non-registry participants, thus supporting the collection of both patient-reported outcomes and data from in-person clinical assessment, in addition to reducing potential selection bias associated with exclusively registry-based recruitment. The steady pace of accrual since study launch suggests support from a motivated patient population as well as suggesting benefit to a study design that supports remote participation and that minimizes burden on participating COG sites.

There is a substantial dearth in our understanding of the long-term impact of cancer treatment in this vulnerable population. To date, the published data have compared late effect prevalence in this at-risk population to non-DS survivors of AL, which disregards the effect of the baseline excess risk for CHC experienced by individuals with DS. For example, the CCSS compared CHC in 154 DS-AL survivors to non-DS survivors matched by diagnosis, age at diagnosis, race/ethnicity, sex, radiation, and chemotherapy exposures, indicating that DS-AL survivors (median age 20 years, range 10–48 years) were at greater risk for serious CHC and ≥ 3 CHC than non-DS AL survivors, with a 25 year CHC cumulative incidence of 83% vs. 69% [49]. Similar results were seen in a smaller cohort of DS-AML survivors [50]. One study evaluated neurocognitive outcomes in DS-AL survivors vs. DS children without AL and found greater deficits in verbal intelligence, spelling, receptive and expressive vocabulary, visual-motor skills, and adaptive function [39]. In a more recent study that compared neurocognitive outcomes between 117 DS controls and 43 DS-AL survivors, differences were observed between the groups in completion rates for executive function and processing speed measures as well as in performance and rating measures of executive function [51].

By addressing this key knowledge gap, the results of ALTE22C1 are expected to inform clinical practice related to both upfront AL treatment and survivorship care. Indications of heightened risk for acute and late-onset chemotherapy-related toxicities specific to the DS population may lead to a change in approach for future clinical trials to mitigate risk for adverse outcomes. The current version of the COG Long-Term Follow-Up Guidelines for Survivors of Childhood, Adolescent, and Young Adult Cancers [52] does not include DS status when assessing treatment-related late effects risk. Robust evidence for excess risk from studies such is this will support added surveillance, leading to early detection, intervention, and a potential reduction in the outcome disparities experienced by this vulnerable population.

## Data Availability

No datasets were generated or analysed during the current study.

## Abbreviations

AL: Acute leukemia
ALL: Acute lymphoblastic leukemia
AML: Acute myeloid leukemia
CCSS: Childhood cancer survivor study
CHC: Chronic health conditions
CI: Confidence interval
COG: Children’s Oncology Group
CTCAE: Common Terminology Criteria for Adverse Events
DS: Down syndrome
LTS: Long-term survivor
ML-DS: Myeloid leukemia of Down syndrome
OR: Odds ratio
SD: Standard deviation
TL: Telomere length
